# A cluster-based cell-type deconvolution of spatial transcriptomic data

**DOI:** 10.1093/nar/gkaf714

**Published:** 2025-07-24

**Authors:** Qingyue Wang, Parth Khatri, Huy Q Dinh, Jian Huang, Yudi Pawitan, Trung Nghia Vu

**Affiliations:** Department of Statistics, University College Cork, Western Rd, T12 XF62 Cork, Ireland; Department of Medical Epidemiology and Biostatistics, Karolinska Institutet, Nobels väg 12A, 17177 Stockholm, Sweden; McArdle Laboratory for Cancer Research, Department of Oncology, School of Medicine and Public Health, University of Wisconsin–Madison, 1111 Highland Ave, WI 53705-227 Wisconsin, United States; McArdle Laboratory for Cancer Research, Department of Oncology, School of Medicine and Public Health, University of Wisconsin–Madison, 1111 Highland Ave, WI 53705-227 Wisconsin, United States; Department of Statistics, University College Cork, Western Rd, T12 XF62 Cork, Ireland; Department of Medical Epidemiology and Biostatistics, Karolinska Institutet, Nobels väg 12A, 17177 Stockholm, Sweden; Department of Medical Epidemiology and Biostatistics, Karolinska Institutet, Nobels väg 12A, 17177 Stockholm, Sweden

## Abstract

Spatial transcriptomics (ST) has emerged as an efficient technology for mapping gene expression within tissue sections, offering informative spatial context for gene activities. However, most current ST techniques suffer from low spatial resolution, where each spatial location often contains cells of various types. Deconvolution methods are used to resolve the cell mixture within the spots, but conventional approaches rely on spot-by-spot analyses, which are limited by low gene expression levels and disregard spatial relationships between spots, ultimately reducing performance. Here, we introduce DECLUST, a cluster-based deconvolution method to accurately estimate the cell-type composition in ST data. The method identifies spatial clusters of spots using both gene expression and spatial coordinates, hence preserving the spatial structure of the tissue. Deconvolution is subsequently performed on the aggregated gene expression of individual clusters, mitigating the challenges associated with low expression levels in individual spots. We evaluate DECLUST on simulated ST datasets from a human breast cancer tissue and two real ST datasets from human ovarian cancer and mouse brain. We compare DECLUST to current methods including CARD, GraphST, Cell2location, and Tangram. The results indicate that DECLUST not only maintains the spatial integrity of tissues but also outperforms existing methods in terms of robustness and accuracy. In conclusion, DECLUST provides an effective and reliable approach for identifying cell-type compositions in ST data.

## Graphical Abstract

**Graphical Abstract:**
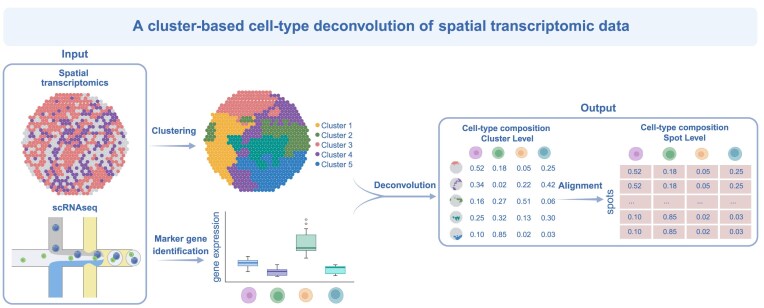

## Introduction

Spatial transcriptomics (ST) enables the acquisition of transcriptome data while preserving the spatial context of cells within tissues, providing novel insights into cellular organization and micro-environmental interactions [1–3]. ST technologies can be broadly categorized into two main types: image-based and RNA-capture-based approaches. Image-based methods, such as *in situ* hybridization (ISH) [4, 5], single-molecule FISH (smFISH) [6], multiplexed error-robust fluorescence *in situ* hybridization (MERFISH) [7], and fluorescent *in situ* sequencing (FISSEQ) [8], provide subcellular resolution and enable direct visualization of individual messenger RNA (mRNA) molecules with high sensitivity. These methods are widely regarded as the gold standard for mRNA quantification. However, these methods are low throughput and the data analysis is complex and challenging. Their application is constrained by the relatively small number of genes, low sensitivity in mRNA detection, and labor-intensive protocols. In contrast, RNA-capture-based methods, such as laser capture microdissection combined with RNA sequencing (LCM + RNA-seq) [9] and array-based platforms like 10× Visium [10], allow for transcriptome-wide profiling across large tissue areas. These methods rely on spatial barcoding and next-generation sequencing to capture gene expression information, offering scalability and broader coverage compared to image-based techniques. However, they are limited by lower spatial resolution, as each capture spot typically contains multiple cells from different cell types [11], complicating the interpretation of cellular heterogeneity and intercellular interactions. The information of cell-type composition at spot level can improve the interpretation of intercellular interactions and support reconstructing the comprehensive landscape of cellular heterogeneity in tissues. This issue can be resolved by cell-type deconvolution based on the gene expression of spots.

Recent cell-type deconvolution methods can be broadly categorized into three main groups: (i) Probabilistic-based methods, which explicitly parameterize data distributions and utilize probability-based inference (e.g. Cell2location [12], RCTD [13], STRIDE [14], and stereoscope [15]); (ii) Matrix factorization-based methods, which use techniques such as non-negative matrix factorization (NMF) and non-negative least squares (NNLS) to infer spot composition (e.g. CARD [16], SPOTlight [17], NMFreg [18], and SpatialDWLS [19]); (iii) Machine learning-based methods, which apply machine learning models to estimate cell-type composition (e.g. Tangram [20] and GraphST [21]). Most deconvolution models do not adequately utilize the spatial information inherent in ST data, thus limiting their accuracy. Some methods such as CARD and GraphST incorporate spatial information in their models; however, they primarily perform deconvolution at the individual spots where inherently low gene expression levels in individual spots in ST data substantially decrease the performance of decomposition methods.

To address these challenges, we developed DECLUST, a cluster-based cell-type deconvolution method with a motivation that deconvolution could be more effective when applied to aggregated gene expression from groups of similar spots, where the signal is inherently stronger. DECLUST identifies spatial clusters of spots by integrating both gene expression profiles and spatial coordinates to preserve the spatial structure of the tissue. Deconvolution is then applied to the aggregated gene expression within each cluster, which overcomes the problem of low expression levels in individual spots.

We compared the performance of DECLUST with four other advanced cell-type deconvolution methods including CARD [16], GraphST [21], Cell2location [12], and Tangram [20]. These methods were selected because of their strong performance in previous benchmarking studies and their frequent use in the recent literature. By including a diverse range of top-performing approaches, we provide a comprehensive comparison across different modeling strategies using both simulated and real datasets.

## Materials and methods

### DECLUST pipeline

**Figure 1.**
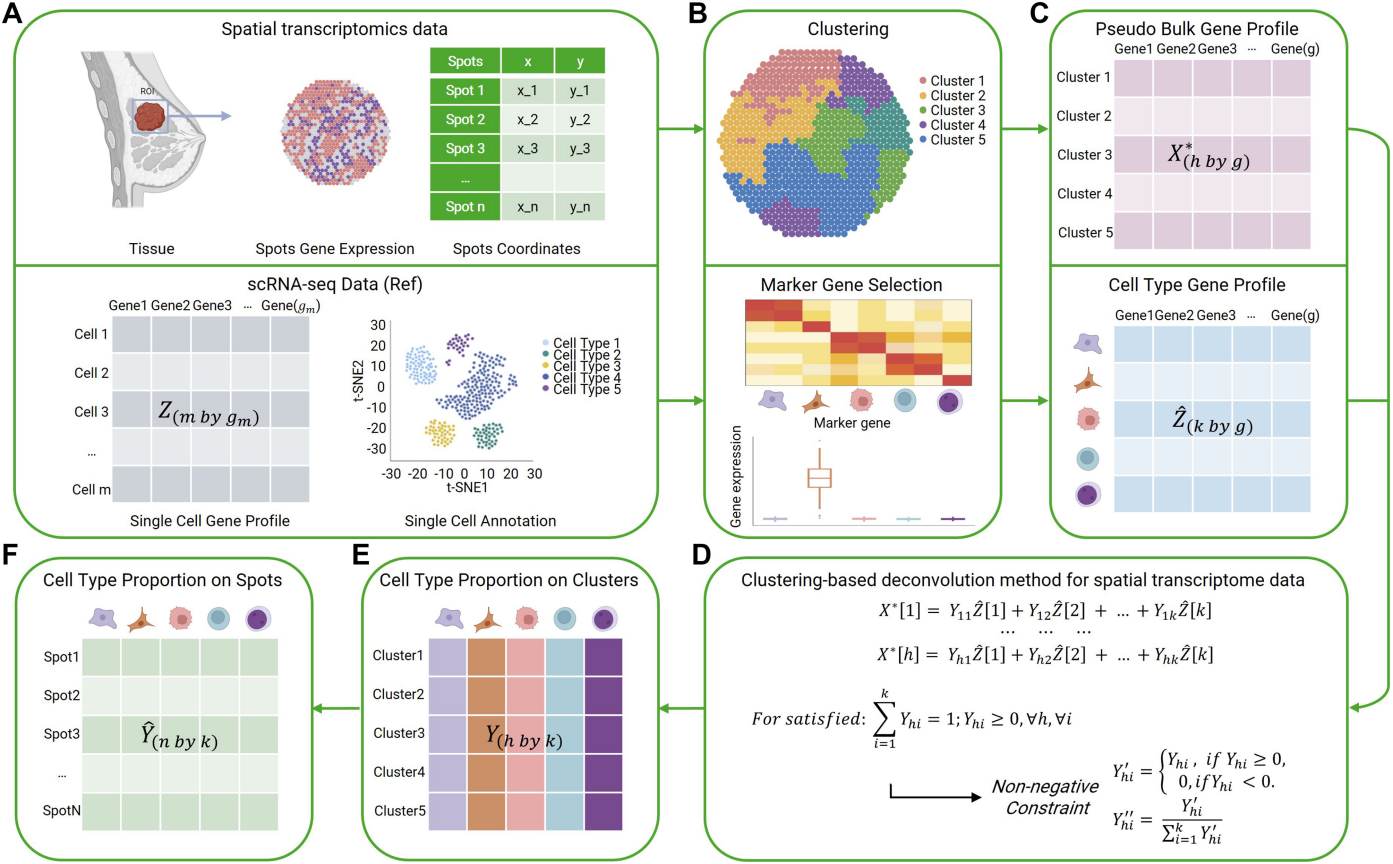
Overview of the DECLUST method. This flowchart illustrates the DECLUST for cell-type deconvolution from ST data. (**A**) Input data of DECLUST consisting of ST data and scRNA-seq data derived from the same tissue sample for reference. (**B**) Data preprocessing involving two primary tasks: (i) clustering spots of the ST data to identify spatial clusters, simultaneously considering both gene expression and spatial information, and (ii) selecting cell-type-specific marker genes from the scRNA-seq data using a two-statistic method. (**C**) Preparation of gene profile matrices for deconvolution, including (i) a pseudo-bulk gene profile matrix for the identified clusters and (ii) a cell-type gene profile matrix for the cell-type-specific marker genes. (**D**) Deconvolution algorithm: the OLS method with a non-negative constraint is employed to estimate the composition of cell-types within each spatial cluster. (**E**) Cluster-level cell-type proportion matrix which is the output of the deconvolution step. (**F**) Spot-level cell-type proportion matrix which is derived from the cluster-level cell-type proportion matrix.

Figure 1 illustrates the DECLUST workflow, from the data input including ST data and reference single-cell RNA sequencing (scRNA-seq) data to the final output of estimated cell-type proportions for individual spots in the ST data. The ST dataset consists of *n* spots, each has a vector of expressions of *g*_*n*_ genes and a 2D coordinate presenting the spatial location of the spot in the tissue image. We denote the gene expression matrix of the ST dataset as *X* (*n* × *g_n_*), including *n* rows of spots and *g*_*n*_ columns of genes. The scRNA-seq data contain a gene expression matrix, denoted as *Z*, including *m* cells and *g*_*m*_ genes. The cells are pre-annotated to belong to *k* distinct cell-types. We retain the top 5000 most variable genes from each dataset, and the overlapping gene set (*g* genes) is kept for downstream analysis.

In the subsequent step, DECLUST deploys a clustering method to identify spatial clusters of spots from the ST data and a two-statistic approach to discover marker genes of each cell-type before applying the ordinary least squares (OLS) algorithm with a non-negative constraint for deconvolution to predict cell-type proportions. The details of these steps will be described in the following sections.

#### Identification of spatial clusters of spots from ST data

This section will present an approach to identify spatial groups of spots based on both gene expression and spatial information, as depicted in Supplementary Fig. S1. Particularly, this method starts with applying the classical hierarchical clustering on gene expression to obtain initial spot clusters. Although the clusters derived based on gene expression contains rich biological signals, the clustering overlooks spatial continuity, leading to potentially fragmented clusters. To address this, the density-based spatial clustering of applications with noise (DBSCAN) [22] is applied to each of the clusters to identify their spatial sub-clusters. These sub-clusters not only capture the underlying spatial structure but also identify isolated spots that do not belong to any dense spatial group, which are treated as potential outliers. The incorrectly clustered spots may arise from the low sensitivity of gene expression profiles of individual spots, which could be improved in the next step. Finally, we generate the set of seeds from these sub-clusters, and input them into the seeded region growing (SRG) algorithm. SRG explicitly grows regions based on both gene expression similarity and spatial proximity. Starting from the seeds identified by DBSCAN, SRG expands the regions in a controlled manner, allowing us to capture subtle spatial patterns while correcting for potential misassignments caused by noisy gene expression or spatial sparsity, resulting in the final clusters for the spots. For deconvolution, gene expression of the spots in each cluster are aggregated into a pseudo-bulk gene profile for the cluster, forming a cluster-gene expression matrix X* including *h* clusters and *g* genes. Next sections describe further details of each step.

##### Hierarchical clustering of spots using gene expression

The hierarchical clustering is applied to the gene expression of spots to identify a set of initial spot clusters. The optimal number of clusters is determined using the elbow method [23, 24], which is based on the plot of the within-cluster sum of squares (WCSS) against the number of clusters. The “elbow” point, where further increases in the number of clusters result in minimal reductions in WCSS, indicates the optimal number of clusters.

The WCSS is calculated as follows:


\begin{eqnarray*}
\text{WCSS} = \sum _{j=1}^{h} \sum _{i=1}^{n_j} \left\Vert x_i^{(j)} - \mu _j \right\Vert ^2
\end{eqnarray*}

where:

- *h* is the total number of clusters.
- *n*_*j*_ is the number of spots in cluster *j*.
-
$x_i^{(j)}$ represents the gene expression profile of the *i*-th spot in cluster *j*.
- μ_*j*_ is the centroid (mean) of cluster *j*, representing the average gene expression profile.
-
$\left\Vert x_i^{(j)} - \mu _j \right\Vert ^2$ measures the squared Euclidean distance between a spot’s gene expression profile and the cluster centroid.

The hierarchical clustering begins with each of the *N* spots treated as an individual cluster. Clusters are iteratively merged based on their proximity using the Ward linkage until the desired number of clusters is reached. The Ward linkage criterion, which minimizes the increase in total within-cluster variance when merging two clusters, is mathematically defined as:


\begin{eqnarray*}
d(A, B) = \frac{|A| + |B|}{T} \cdot \left\Vert \text{centroid}_A - \text{centroid}_B \right\Vert ^2
\end{eqnarray*}

where:

- *d*(*A*, *B*) is the distance between clusters *A* and *B* after merging.
- |*A*| and |*B*| are the sizes of clusters *A* and *B*, respectively.
- *T* = |*A*| + |*B*|, the total number of spots in both clusters.
- ‖centroid_*A*_ − centroid_*B*_‖^2^ is the squared Euclidean distance between the centroids of clusters *A* and *B*.

The clusters identified by the hierarchical clustering provide a structure of the spots based on gene expression, but neglect the spatial relationships between spots. In the next section, for each cluster, DBSCAN algorithm will be applied on its spots to explore spatial sub-clusters based on their spatial relationship. Using these sub-clusters, a set of the pre-selected seeds will be collected for the SRG method at the last step.

##### Spatially sub-clustering of spots using DBSCAN and seed selection

To identify spatial sub-clusters of each initial cluster in the previous step, DBSCAN is applied to the coordinates of spots within the cluster. DBSCAN requires two parameters: ε, the maximum Euclidean distance between spots within the same sub-cluster, and minPts, the minimum number of spots required to form a sub-cluster. In practical implementation of DECLUST for ST data, we select ε = 4 and minPts = 8.

Selection of seeds for the SRG method is based on the sub-cluster size:

- For a small sub-cluster with <5% of the total spots, none spots of the sub-cluster are designated as seeds.
- For a sub-cluster containing no <5% of the total spots, 50% of the spots in the sub-cluster are randomly selected as seeds, excluding the spots at the boundary of the tissue image.

Finally, each detected seed is assigned with a label by its corresponding sub-clusters. The set of seeds with their labels is inputted into the SRG algorithm in the next section to finalize the spot clusters for cell-type deconvolution.

In DECLUST, the choice of the parameters is empirically determined through extensive testing across multiple datasets. If the proportion of seeds from spots is too high, the spots are highly depending on the sub-cluster label which is determined by the hierarchical clustering in the previous step. In contrast, if the proportion too small, it means many spots are uncertain to belong to their current sub-clusters, which are later determined via the updating process by gene expression distance and/or their neighbors.

##### Seeded region growing method for spot segmentation to identify final spot clusters

In this section, we apply the SRG, an efficient segmentation method in image processing, to construct the spot clusters based on both the spatial relationship and gene expression similarity between spots. The algorithm utilizes the set of seeds and their labels identified from the DBSCAN in the previous step to construct clusters based on the minimum distance between spots and the seeds. To facilitate the search for the spot with minimum distance to the seeds, SRG constructs a sequentially sorted list (SSL). SRG defines an SSL as the set of spatial neighboring spots of the current seeds. The SSL is defined as a set of spatial neighboring spots of the current seeds, and is sorted by the gene-expression distance between the spots and their neighboring seeds [25].

SRG expands the seeds’ regions iteratively based on the distance of neighboring spots stored in the SSL. At each iteration, the closest spot *p* is removed from the top of the SSL, then labelled based on its neighbors. Next, its unlabeled neighbors are added to the SSL. The process repeats until every spot is assigned with a label. The label of spot *p* is assigned based on the following criteria:


\begin{eqnarray*}
&& L(p) = \\ && \left\lbrace \begin{array}{@{}l@{\quad }l@{}}L(q) & \text{if } L(q) = L(r) \text{ for all neighboring spots } q, r \\ L_{\text{nearest}} & \text{if neighboring spots have different labels} \end{array}\right.
\end{eqnarray*}

where *L*(*p*) is the label assigned to the spot *p*, and *L*_nearest_ refers to the label of the closest neighboring region in terms of the gene expression profile.

The details of the SRG algorithm can be found in the related study [25]. SRG not only efficiently integrates spatial relationship and gene expression similarity of spots to generate clusters, but also automatically determines the number of clusters, enhancing the the overall segmentation accuracy and quality of ST data.

#### Discovery of marker genes from scRNA-seq data

We apply a two-statistic approach outlined in a recent study [26] to identify cell-type specific genes for *k* cell-types from scRNA-seq reference data, then use them as marker genes for the deconvolution step. We consider a cell-type specific marker gene if the expression of the gene is highly up-regulated in one cell-type, but its expression in the remaining cell-types is not statistically different. To do this, we employ two statistics: a robust *t*-test (T1) and a chi-square test (T2). T1 determines whether a given cell-type differs significantly from all others, while T2 assesses whether the remaining cell-types exhibit similar expression levels. A gene is considered cell-type-specific if T1 is large, indicating a statistically significant difference from other cell-types, and T2 is small, indicating no significant difference among the remaining cell-types. Statistical significance is controlled using *P*-values, with thresholds set at *P* for the T1 < 0.05 and *P* for the T2 > 0.05. Top *t* genes by t-statistics are selected as marker genes for each cell-type to build a reference matrix. In implementation, we keep top *t* = 10 genes for individual cell-types. The reference matrix $\hat{Z}(k \times g$) is constructed from *k* cell-types and *g* marker genes. For a marker gene *j* and cell-type *i*, the value of $\hat{Z}_{i,j}$ is calculated by averaging gene expression of the marker gene across all single cells belonging to the cell-type in the single-cell data reference.

#### Cell-type deconvolution using ordinary least squares

The primary objective of the deconvolution task is to estimate the cell-type proportion matrix *Y*(*m* × *j*) based on the cluster-gene profile matrix X*(*h* × *g*) and cell-type gene profile matrix $\hat{Z}(k \times g$), which are identified in the previous steps. The problem is formulated as a system of linear equations:


\begin{eqnarray*}
X^*[1] &=& Y_{11} \hat{Z}[1] + Y_{12} \hat{Z}[2] + \dots + Y_{1k} \hat{Z}[k], \\ X^*[2] &=& Y_{21} \hat{Z}[1] + Y_{22} \hat{Z}[2] + \dots + Y_{2k} \hat{Z}[k], \\ &\vdots \\ X^*[h] &=& Y_{h1} \hat{Z}[1] + Y_{h2} \hat{Z}[2] + \dots + Y_{hk} \hat{Z}[k].
\end{eqnarray*}

where:

- X*[*h*] represents the gene expression profile for the *h*-th spatial cluster. Each row in the matrix X* corresponds to a different spatial cluster, capturing the expression levels of a set of genes for that cluster.
-
$\hat{Z}[k]$ represents the gene expression profile for the *k*-th cell-type. Each row in the matrix $\hat{Z}$ corresponds to a different cell-type, describing how strongly each gene is expressed in that particular cell-type.

The cell-type proportions for each cluster are estimated using OLS with a non-negative constraint. The estimates are obtained as follows.


\begin{eqnarray*}
\begin{aligned} Y[1] &= \left( \hat{Z}^T \hat{Z} \right)^{-1} \hat{Z}^T X^*[1], \\ Y[2] &= \left( \hat{Z}^T \hat{Z} \right)^{-1} \hat{Z}^T X^*[2], \\ &\vdots \\ Y[h] &= \left( \hat{Z}^T \hat{Z} \right)^{-1} \hat{Z}^T X^*[h]. \end{aligned}
\end{eqnarray*}

where each cell-type proportion vector *Y*[*i*] of cluster *i* is constrained by:


\begin{eqnarray*}
\sum _{j=1}^{k} Y_{ij} = 1, \quad Y_{ij} \ge 0, \quad \forall i, \forall j.
\end{eqnarray*}

To keep the non-negative constraint, each *Y*_*ij*_ is adjusted as follows:


\begin{eqnarray*}
Y^{\prime }_{ij} = \left\lbrace \begin{array}{@{}l@{\quad }l@{}}Y_{ij}, & \text{if } Y_{ij} \ge 0, \\ 0, & \text{if } Y_{ij}\,<\, 0. \end{array}\right.
\end{eqnarray*}

Then, the vector *Y*′[*i*] is normalized to keep the sum of cell-type proportions equal 1:


\begin{eqnarray*}
Y^{\prime \prime }_{ij} = \frac{Y^{\prime }_{ij}}{\sum _{j=1}^{k} Y^{\prime }_{ij}}.
\end{eqnarray*}

The resulting matrix *Y*, representing cell-type proportions at the cluster level, is then expanded into a matrix $\hat{Y}(n \times k$) to achieve the cell-type proportions at spot level.

### Datasets

#### Real datasets

Two real ST datasets generated by the 10x Genomics Visium Spatial Gene Expression platform [27] are used in this study.

##### Mouse brain ST dataset

Fresh frozen coronal sections from a male C57BL/6 mouse (>8 weeks old) were prepared using the 10x Genomics Visium Spatial Gene Expression technology. Tissue sections of 10 µm thickness were stained with Hematoxylin and Eosin (H&E) and imaged using a Nikon Ti2-E microscope. Spatial gene expression libraries were sequenced on an Illumina NovaSeq 6000 with paired-end reads (28 × 90 bp), achieving an average sequencing depth of 115 740 reads per spot. There were a total of 2698 spots, the median of 27 198 UMI counts, and 5798 genes were detected per spot. This dataset is used to assess the accuracy of predicted cell-type proportion and how well the cell-type deconvolution methods keep the spatial relation between spots through cross-validation.

##### Single-cell reference data for the mouse brain ST dataset

The gene expression of this scRNA-seq dataset was derived from the primary visual cortex of 6–8-week-old C57BL6/J mice. The dataset, generated using the inDrops platform, included data from 16 188 cells and 25 187 genes, covering eight major cell-types. The details of this dataset can be found in the original article [28, 29].

##### Human ovarian cancer ST dataset

Formalin-fixed, paraffin-embedded (FFPE) tissue sections (5 µm) from a case of serous papillary carcinoma were sourced from iSpecimen (Block 108906). Sections were processed using the Visium platform, stained, and imaged using a brightfield camera at 20× magnification. Libraries were sequenced on an Illumina NovaSeq 6000 with paired-end reads (28 × 75 bp), with a sequencing depth averaging 63 836 reads per spot. There were a total of 3455 spots, the median of 28 927 UMI counts and 7621 genes were detected per spot. We utilize this dataset to investigate the preservation of spatial pattern of cell-type marker genes from ST data.

##### Single-cell reference data for human ovarian cancer ST dataset

The gene expression of this dataset consisted of 26 906 genes and 18 299 cells, generated by 10× genomics single-cell 5’ RNA sequencing for nine patients with high-grade serous ovarian carcinoma (HGSOC). The dataset captured the tumor’s cellular heterogeneity, including epithelial tumor cells, tumor-infiltrating immune cells, and various stromal components. More information about this dataset can be referred to its original study [30, 31].

#### Simulated ST datasets

In this study, we utilize the real human breast cancer ST dataset to simulate ST data for evaluation of the deconvolution methods. This dataset involved a Visium Spatial Gene Expression experiment on fresh frozen Invasive Ductal Carcinoma breast tissue (AJCC/UICC Stage IIA, ER-positive, PR-negative, and Her2-positive), obtained from BioIVT Asterand. The tissue was 10 µm thick, placed on Visium Gene Expression Slides, and imaged using a Nikon Ti2-E microscope. Sequencing was performed on an Illumina NovaSeq 6000 with a configuration of 28 × 120 bp and a depth of 150 392 reads per spot. A total of 3798 spots were detected, with a median of 20 762 UMI counts per spot and a median of 6026 genes per spot.

##### Simulation 1.

We first apply DECLUST on the dataset to predict cell-type compositions of spots, then use them to simulate the gene expression of spots using single cell reference data. The single-cell transcriptomics dataset comprises 100 064 cells across nine major cell types, collected from 26 primary tumors representing three breast cancer subtypes: 11 ER+, 5 HER2+, and 10 TNBC patients. It contains expression data for 29 733 genes [32, 33]. Specifically, for each spot, we use the multinomial distribution to generate cell counts of each cell-type according to the cell-type proportions by DECLUST and the total number of cells in the spot. We fix the number of cells per spot to 10, which is in the range value of the number of cells in the spots from 10× Visium technology. Next, we randomly selected single cells from the single-cell reference dataset using generated cell counts for each cell-type. Finally, the gene expression profiles of the selected cells are aggregated to create a simulated gene expression profile of the spot. We use the same spot coordinates of the real ST data for the simulated ST dataset. Thus, the simulated ST dataset preserves the spatial structure observed in the original data while controlling cell-type proportions of spots.

We generate ten simulated datasets by repeating the procedure, allowing us to assess the variability of the deconvolution methods. The simulation 1 replicates are used to evaluate the variability of deconvolution methods, which is discussed in “Results” section.

##### Simulation 2.

We have added ligand–receptor (LR) interactions in addition to Simulation 1 framework for generating ST datasets, which mimic real ST data. We used **BayesSpace** [34], a method that incorporates both gene expression and spatial coordinates to detect spatial domains, to generate spatial clusters for real breast cancer ST dataset. Then, cell-type proportions are inferred for each cluster using **CIBERSORT** [35], a widely used reference-based bulk deconvolution algorithm.

To mimic cell-type heterogeneity and co-localization within each cluster, we select a pair of dominant cell types and simulate enrichment for the spots of the cluster using LR interactions with a high probability, which are inferred from **CellChat** [36]. To make the LR enrichment specific for a pair of cell types, we select only single cells highly expressed in both ligand and receptor from the single cell reference dataset for the simulation. Specifically, single cells satisfying the expression of both ligand and receptor in the third quartile (top 25%) are used. In Simulation 2, we select two clusters for enrichment, including:

- Cluster 4, guided by the *CXCL12–CXCR4* interaction, we enriched *CAF* and *Plasmablast* cells.
- Cluster 7, guided by *TNF–TNFRSF1A*, we enriched *Myeloid* and *Endothelial* cells.

The spatial expression patterns of the selected LR pairs are visualized in Supplementary Fig. S2 to illustrate their role in guiding cell-type-specific enrichment in Simulation 2.

### Competing cell-type deconvolution methods

To benchmark our model, we compared it against four established cell-type deconvolution methods that utilize a variety of computational approaches. These methods, widely adopted for both bulk RNA-seq and ST data, leverage different strategies such as matrix factorization, probabilistic inference, graph-based learning, and deep learning. Each of these methods aims to decompose mixed cellular signals into distinct cell-type-specific profiles. A detailed summary of these methods can be found in Supplementary Table S1.

**CARD** employs non-negative matrix factorization to infer cell-type proportions by using highly variable genes and single-cell RNA-seq data as reference profiles. It incorporates spatial information of ST into deconvolution, improving the accuracy of both proportion estimates and spatial maps of cell distributions.

**Cell2location** adopts a probabilistic model with variational Bayesian inference, assuming gene expression follows a negative binomial distribution. It relies on scRNA-seq reference data for estimating cell-type-specific signatures and models the spatial expression as a combination of these signatures.

**GraphST** utilizes a graph neural network-based framework, constructing a spatial graph from highly variable genes and applying self-supervised contrastive learning. This method aligns spatial gene expression with reference scRNA-seq data, capturing local spatial patterns and producing deconvolved cell-type distributions.

**Tangram** illustrates a deep learning model to map scRNA-seq or single-nucleus RNA sequencing (snRNA-seq) data onto ST data. By maximizing the correlation between shared gene expression across datasets, Tangram estimates cell-type proportions while maintaining spatial coherence.

### Performance metrics for simulation study

In each simulated dataset, the true cell-type proportions of a spot is calculated by the ratio between cell-type’s population and the total number of cells in that spot. We denote the true cell-type proportion matrix of the spots as *S*. After obtaining the estimated cell-type proportion matrix $\hat{Y}$ from a deconvolution method, we assess its performance by computing the Root Mean Square Error (RMSE) between $\hat{Y}$ and *S* as follows:


\begin{eqnarray*}
\text{RMSE} = \sqrt{\frac{1}{g} \sum _{j=1}^{g} \left( \hat{Y}_{ij} - S_{ij} \right)^2}
\end{eqnarray*}

where $\hat{Y}_\mathrm{ij}$ and *S*_ij_ are the estimated and true cell-type proportion of the *i*th spot for the *j*th cell-type, respectively. For the calculation of Mean Square Error (MSE), we exclude the squared root from the formula.

### Cross-validation for real ST data

We aim to use cross-validation to evaluate performances of the deconvolution methods in both cell-type proportion estimation accuracy and in the preservation of spatial relation of spots. To do cross-validation, we first randomly divide the spots in each cluster to 10-folds, thus the spots in each fold are independent and spatially non-adjacent. Then, each fold in turn selected as a test set, the remains are used for training set, across clusters. Next, a deconvolution model is applied to the training set to estimate cell-type proportions of individual spots. For the spots in the test set, the cell-type proportions are predicted by the average values of the neighboring spots from the training set.

The rationale behind this is that the molecular profiles of neighboring spots in ST dataset are highly correlated, as shown in Fig. 3B. Therefore, we can use the predicted cell-type proportions for the test set as the baseline for comparison of deconvolution methods. The result of a good deconvolution method is expected to keep well the spatial relation between spots. Of note, our intention with Fig. 3B is not to claim the existence of local correlation as a novel finding. It is a model-independent base performance that can be utilized as a target to evaluate deconvolution methods in real ST data where ground-truth proportions are unavailable. Similar to DECLUST, other methods including CARD and GraphST do incorporate spatial information into their models. So, the information does not give DECLUST a special advantage.

**Figure 2.**
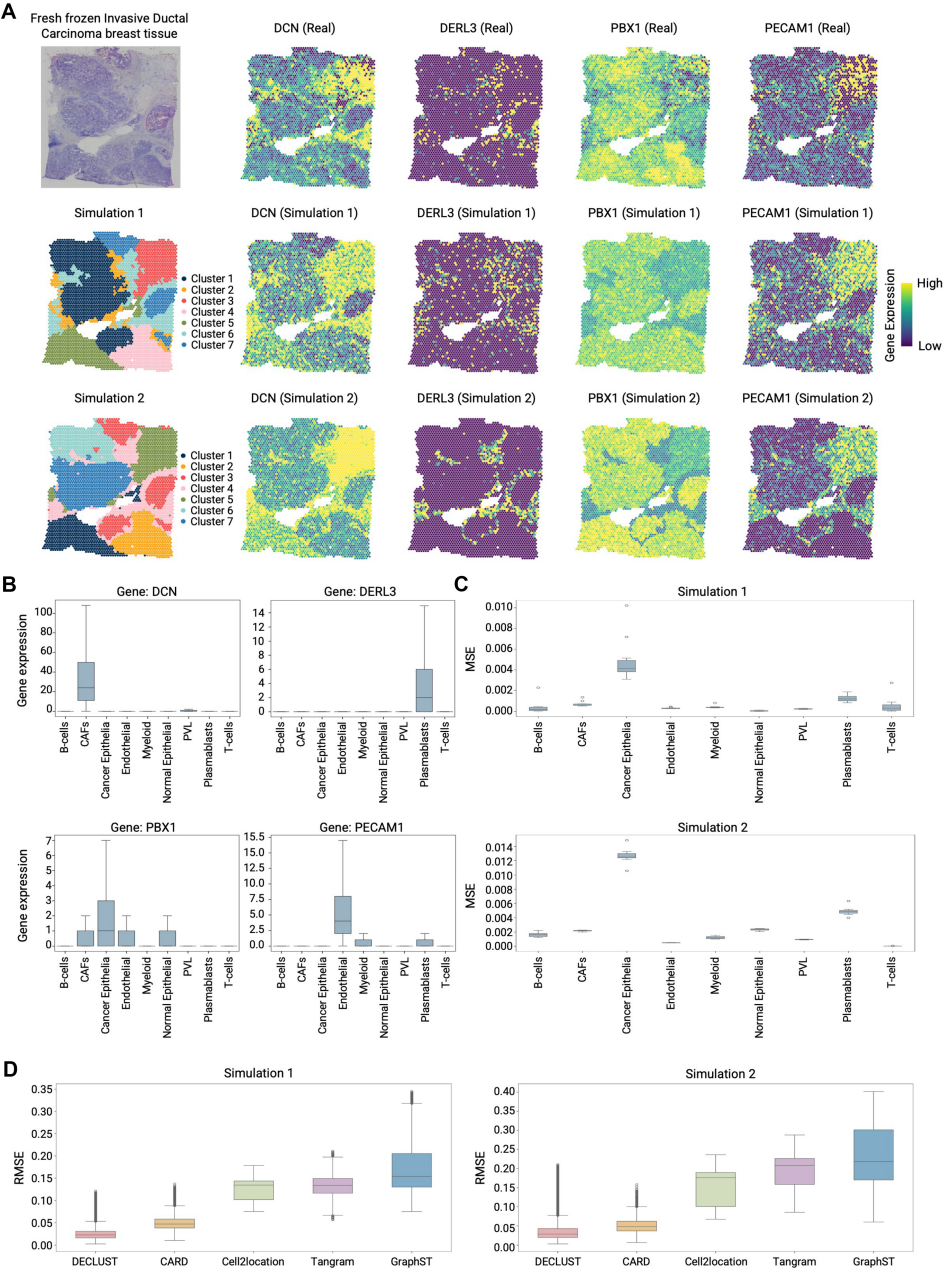
Comparison of DECLUST with other cell-type deconvolution methods in simulated datasets. (**A**) The similarity between the simulated and real ST datasets from a fresh frozen invasive ductal carcinoma breast tissue. The top left panel displays the original H&E-stained image of the tissue. The middle and bottom left panels show clustering results generated by DECLUST in Simulation 1 and Simulation 2, respectively, each revealing the spatial distribution of seven clusters. The remaining panels present the expression patterns of four marker genes including DCN, TYROBP, PBX1, and PECAM1 from four most dominant cell-types: CAFs, myeloid cells, cancer epithelia, and endothelial cells, respectively. The top row shows gene expression heatmaps from the real ST data, while the second and third rows display Simulation 1 and Simulation 2, respectively. (**B**) Box plots representing the expression levels of the top cell-type-specific marker genes across the four dominant cell types. (**C**) Performance of DECLUST simulation study by MSE calculated across 10 (C) replicate datasets of Simulation 1 (top) and Simulation 2 (bottom). (**D**) Comparison of DECLUST against other advanced deconvolution methods including CARD, Cell2location, Tangram, and GraphST in Simulation 1 (left) and Simulation 2(right). The box plots present the root mean squared error (RMSE) of all spots derived from the deconvolution methods. The box plots display the first and third quartiles, with the median as a horizontal line and whiskers extending to 1.5 times the IQR.

**Figure 3.**
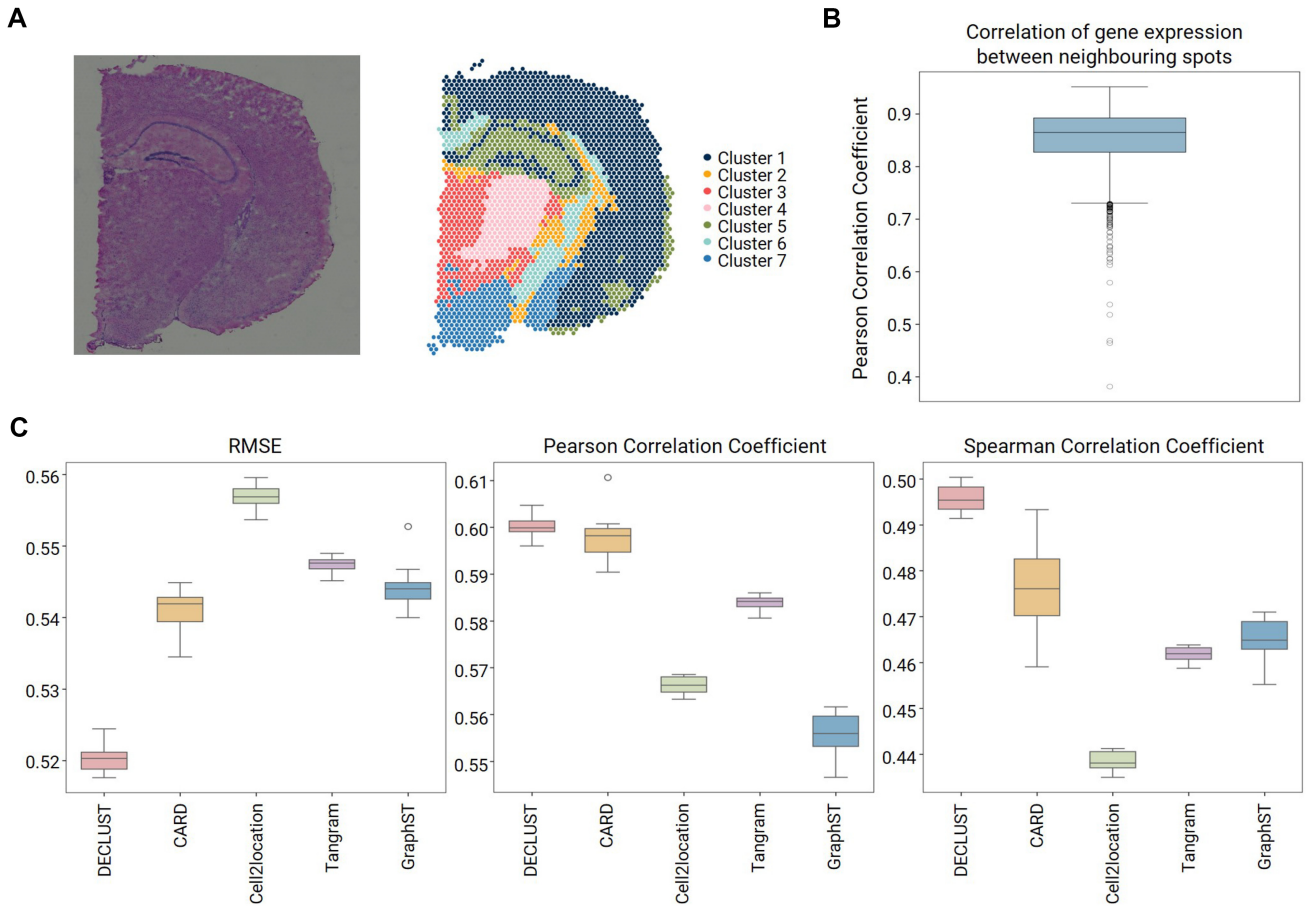
Cell-type deconvolution results for the real mouse brain ST data. (**A**) DECLUST clustering results reflect the observed structural features of the mouse brain coronal section. For example, cluster 5 accurately captures the region within the dark contour of the brain image, corresponding to the hippocampal region. (**B**) PCCs between spots and their neighbors. The *x*-axis presents the spot ID and the *y*-axis indicates the value of PCC. (**C**) The 10-fold cross-validation results for five cell type deconvolution methods are presented in terms of RMSE, PCC, and SCC.

Subsequently, we reconstruct the gene expression profile of the spot in the test set using a convolution process. The convolution is performed for the predicted proportions of cell types and the average gene expression across all cells of the cell types from the single-cell reference data. Finally, the performance of the deconvolution methods are assessed by comparing these convoluted gene expression profiles with the observed values at spot level in the test set. We use the following metrics to assess metrics to assess the cross-validated performance of the deconvolution methods.

**Root Mean Square Error**


(1) \begin{eqnarray*}
\text{RMSE} = \frac{1}{n} \sum _{i=1}^{n} \sqrt{\frac{1}{g} \sum _{j=1}^{g} (y_{ij} - \tilde{y}_{ij})^2}
\end{eqnarray*}

where *n* is the number of spots in the test set, *g* is the number of genes, *y*_*ij*_ is the observed of gene *j* on spot *i*, $\tilde{y}_{ij}$ is the predicted expression level of gene *j* on spot *i*.

**Pearson Correlation Coefficient**


(2) \begin{eqnarray*}
\text{PCC} = \frac{1}{n} \sum _{i=1}^{n} \frac{\sum _{j=1}^{g} (y_{ij} - \bar{y}_i)(\tilde{y}_{ij} - \bar{\tilde{y}}_i)}{\sqrt{\sum _{j=1}^{g} (y_{ij} - \bar{y}_i)^2} \cdot \sqrt{\sum _{j=1}^{g} (\tilde{y}_{ij} - \bar{\tilde{y}}_i)^2}}
\end{eqnarray*}

where the symbols defined are the same as those for calculating the RMSE value, added $\bar{y}_i$ is the observed mean expression level in the spot *i*, $\bar{\tilde{y}}_i$ is the predicted mean expression level in the spot *i*.

**Spearman Correlation Coefficient**


(3) \begin{eqnarray*}
\text{Spearman} = \frac{1}{n} \sum _{i=1}^{n} \left( 1 - \frac{6 \sum _{j=1}^{g} (R_{y_{ij}} - R_{\tilde{y}_{ij}})^2}{g(g^2 - 1)} \right)
\end{eqnarray*}

where $R_{y_{ij}}$ and $R_{\tilde{y}_{ij}}$ represent the rankings of the *i*th site and the *j*th gene in the observed and predicted gene expression levels, respectively.

## Results

### Description of DECLUST methodology

The pipeline of DECLUST to estimate cell-type composition from ST data is presented in Fig. 1. DECLUST highlights the value of spatial information for enhancing deconvolution performance. The motivation of DECLUST stems from the idea that deconvolution could be more effective when applied to aggregated gene expression from groups of similar spots, where the signal is inherently stronger. The key steps of DECLUST include (i) clustering spots using both gene expression profiles and spot spatial coordinates; (ii) identifying cell-type-specific marker genes for reference from single-cell transcriptomics; and (iii) estimating cell-type proportions using the gene expression profiles of the clusters and cell-type-specific marker genes. Although this strategy sacrifices some spatial granularity, it enhances signal robustness in regions with sparse expression. Importantly, the clusters are both transcriptionally similar and spatially coherent, consistent with prior observations that neighboring cells in tissues tend to form biologically meaningful domains [37].

The input for DECLUST in Fig. 1A includes an ST dataset for deconvolution and scRNA-seq data from the same tissue as a reference. The ST dataset consists of gene expression profiles and spatial coordinates of spots, and the scRNA-seq reference provides gene expression profiles along with corresponding cell-type annotations for individual cells. DECLUST starts with identification of spatial clusters of spots from the ST data using its proposed clustering method based on both gene expression profiles and spot spatial coordinates (Fig. 1B). Marker genes of each cell type are also discovered from the scRNA-seq data using a two-statistic approach. The outputs of these methods are presented in Fig. 1C, including two matrices: a pseudo-bulk gene profile matrix (X*) of spatial clusters of spots and a cell-type gene profile matrix ($\hat{Z}$) derived from the scRNA-seq reference. Subsequently, the matrices are entered into the OLS algorithm [38, 39] with a non-negative constraint to estimate the cell-type proportion of individual spatial clusters (Fig. 1D). The OLS output is presented by a matrix *Y* in Fig. 1E, which is later used to achieve the estimated cell-type proportions for individual spots, matrix $\hat{Y}$, in Fig. 1F.

### DECLUST achieves high accuracy in simulated ST data

The simulated ST datasets to evaluate DECLUST are generated based on a real human breast cancer ST data produced by 10× Visium [27]. Figure 2A shows that the simulated ST data closely resemble the real ST data and effectively preserve the original tissue structure. In the left-most column of Fig. 2A, the image of the real ST data is shown at the top, while the next two images present the spot clusters of the simulated ST data in Simulation 1 and Simulation 2. The remaining columns of Fig. 2A illustrate the similarity between the simulated and real ST data by comparing the spatial expression patterns of the top marker genes for four of the most dominant cell types: CAFs, myeloid cells, cancer epithelia, and endothelial cells, arranged from left to right. Because the real ST data usually lacks of the cell-type annotations from the ground truth, marker genes can serve as proxies for evaluating the spatial distribution of cell types [40–43]. The top marker genes, shown in Fig. 2B, are identified from a publicly available scRNA-seq breast cancer dataset [32], satisfying that they are highly expressed in one cell type but not showing statistically significant differences in the remaining cell types.

Figure 2C presents the performance of DECLUST in estimation of cell-type proportions across 10 replicate datasets of Simulation 1 (top) and Simulation 2 (bottom). The cancer epithelial, the most abundant cell type, has the largest mean squared error (MSE) of 0.013 in Simulation 2, while the MSEs of all other cell types are <0.004, indicating the high accuracy of DECLUST. To further illustrate the accuracy of DECLUST in predicting cell-type proportions, Supplementary Figs S4 and S5 provide scatter plots comparing the true cell-type proportions with the predicted proportions for each method across all 10 replicates in Simulation 1 and Simulation 2, respectively. The close alignment of points along the diagonal line in the DECLUST and CARD methods demonstrates their superior performance, while other methods, particularly GraphST, show greater variability and divergence from the true proportions. This visualization underscores the strong predictive accuracy of DECLUST across various simulated datasets.

Figure 2D shows that DECLUST also outperforms existing deconvolution methods including CARD, GraphST, Cell2location, and Tangram in the simulated ST data. This figure presents the results of Simulation 1 (left) and Simulation 2 (right). As shown in the figure, DECLUST achieves the lowest RMSE compared to the other methods in both simulations, although its RMSE values in Simulation 2 are higher then Simulation 1. CARD follows as the runner-up with an RMSE slightly higher than DECLUST. The remaining methods—Cell2location, Tangram, and GraphST—show progressively higher RMSE values, with GraphST performing the worst.

Furthermore, the results of DECLUST are less varied across simulation replicates with the narrow interquartile range (IQR) in the box plots than the other methods, Supplementary Fig. S3. The narrow IQR indicates DECLUST’s reliability for cell-type deconvolution from ST data, producing results with consistent accuracy.

### DECLUST performs well against other methods in the analysis of the real mouse brain ST data

Next we apply cross-validation to evaluate performance of DECLUST in the analysis of a real ST dataset of a mouse brain produced by 10× Visium [27]. The corresponding scRNA-seq dataset for the reference is collected from a recent study [28, 29], consisting of eight cell types, which will be used for deconvolution. DECLUST suggests seven clusters for the mouse brain ST data, Fig. 3A, closely aligning with the observed image structure. For example, the hippocampal region inside temporal lobe, surrounded by a dark blue contour in the brain image at the left panel, is well captured by cluster 5 discovered by the DECLUST’s clustering method at the right panel.

The true cell-type proportions of individual spots are usually not available in a real ST data. However, we expect a high correlation of molecular profiles between spatially neighboring spots; this can be utilized to evaluate deconvolution methods. The neighbor of a spot is one of eight spots spatially adjacent with that spot in the tissue image. Indeed, Fig. 3B shows that the gene expression profiles of spatially neighboring spots are highly correlated. In this figure, the boxplot represents the distribution of PCCs between the observed gene expression of a spot and the predicted value, which is calculated by averaging the gene expression of its eight neighboring spots. The average PCC across all spots is 0.85, indicating that the molecular profile of a spot can be well approximated from its neighbors. While this spatial correlation is biologically expected and has also been incorporated into models such as CARD and GraphST, DECLUST differs in that it explicitly aggregates spatially coherent spots prior to deconvolution. This strategy improves robustness by enhancing the signal-to-noise ratio and results in smoother transitions across spatial domains, which is reflected in the correlation-based performance comparison. We apply this idea to cell-type proportions as the basis for comparing deconvolution methods: A good deconvolution method should preserve the spatial correlation between neighboring spots.

The results of 10-fold cross-validation are evaluated using three performance metrics: RMSE, PCC, and Spearman Correlation Coefficient (SCC), as shown in Fig. 3C. Consistent with the simulation study results, DECLUST and CARD outperforms the remaining methods in all performance metrics. Specifically, DECLUST obtains the median RMSE of 0.525 significantly higher than the follow-up method CARD (median RMSE = 0.542, *P*-value=9.3 × 10^−13^). Similar result is observed for SCC, where DECLUST has higher the median *r* = 0.495 in comparison with *r* = 0.475 of CARD (*P*-value=1.2 × 10^−5^). The PCC results of DECLUST (median *r* = 0.60) are comparable with those of CARD (median *r* = 0.598). For the remaining deconvolution methods, Cell2location generally shows the poorest performance compared to Tangram and GraphST.

### DECLUST effectively preserves spatial pattern of cell-type marker genes in the real human ovarian cancer ST data

We now utilize an ST dataset of a human ovarian cancer tissue [27] to compare the deconvolution methods on their preservation of spatial pattern of cell-type marker genes. Of note, as discussed in the simulation study, cell-type marker genes can serve as proxies for evaluation of the spatial distribution of cell types. Among the 22 different cell types from the single-cell reference dataset of human ovarian cancer [30, 31], we selected three different cell types for evaluation based on their sizes: tumor cells (*n* = 5438 cells, the most prevalent cell type), mesangial cells (*n* = 338 cells, a moderately abundant cell type), and chondrocytes (*n* = 4 cells, a rare cell type).

**Figure 4.**
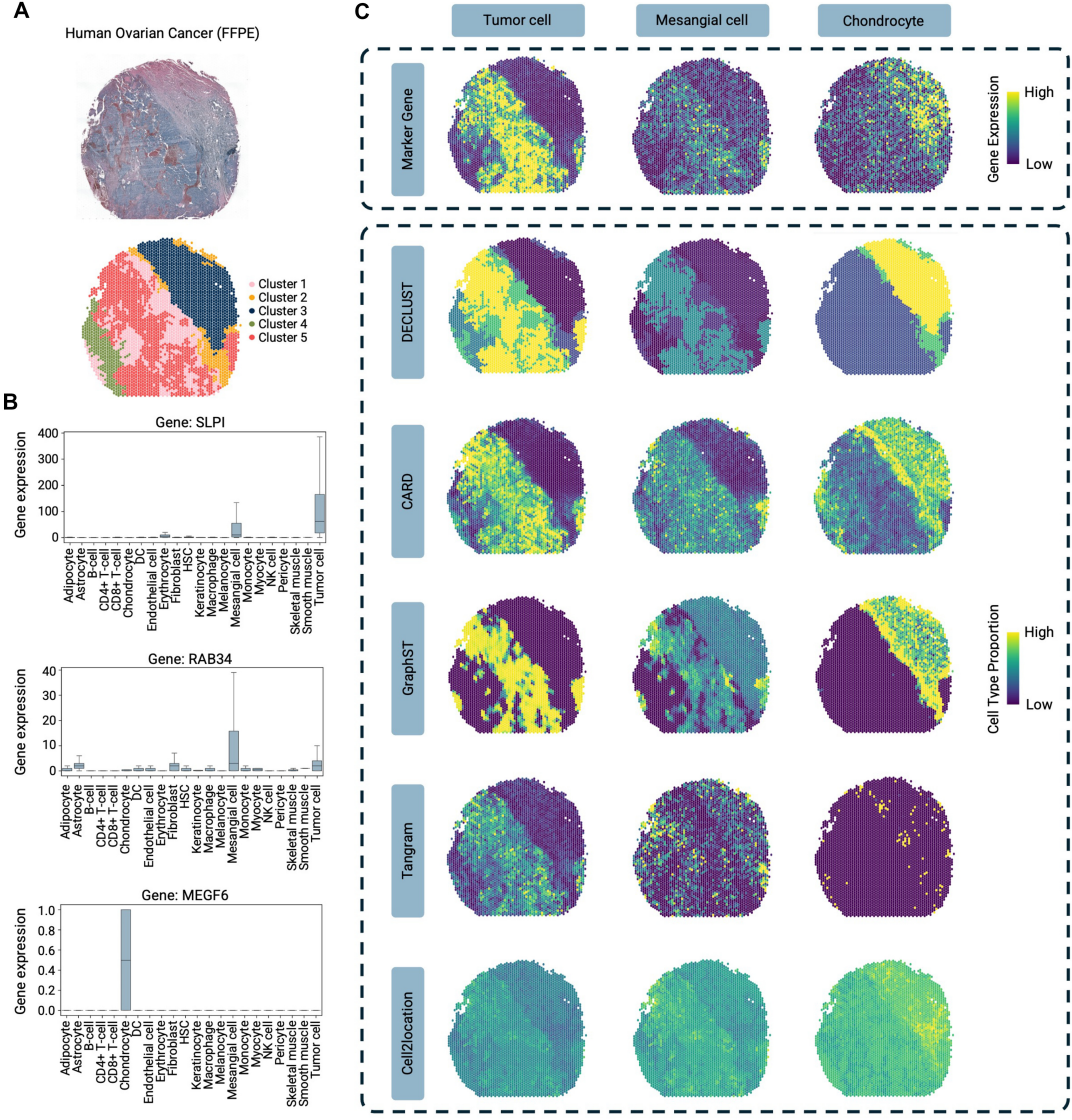
Cell-type deconvolution results for the real human ovarian cancer ST data. (**A**) Clustering results of DECLUST for the ST data of a human ovarian cancer tissue (FFPE). The top image shows the original H&E-stained FFPE ovarian cancer tissue, while the bottom panel shows five clusters discovered by DECLUST. (**B**) Box plots representing the expression levels of the marker genes including SLPI, RAB34, and MEGF6. (**C**) Spatial distribution of expression of marker genes and cell-type proportions in the human ovarian cancer ST data. The top box shows observed expression of three marker genes: SLPI, RAB34, and MEGF6, the corresponding to the results from DECLUST and other methods for tumor cells, mesangial cells, and chondrocyte in the bottom box.

Figure 4A shows the image of the human ovarian cancer (top) and distribution of five clusters discovered by DECLUST (bottom). Figure 4C displays the spatial pattern of the three selected cell types from the ovarian cancer tissue, each in one column. For each column, the heatmap in the first row displays the observed gene expression of the single marker gene of the cell type discovered by DECLUST (Fig. 4B). The corresponding heatmap at the second row shows the proportion of that cell type predicted by DECLUST. The high concordance between two heatmaps across three cell types demonstrates the DECLUST’s ability to accurately capture the spatial patterns of the cell-type marker genes. We further investigated the spatial expression of the top 5, 10, 25, and 50 marker genes for selected cell types from the real ST data. For each case, we selected the corresponding number of top marker genes based on prior knowledge, summed up their normalized expression values across spots, and plotted the resulting aggregated expression as a heatmap. Each heatmap thus represents the spatial expression pattern derived from a distinct set of marker genes. As shown in Supplementary Fig. S6, tumor cells, mesangial cells, and chondrocytes exhibit consistent and coherent spatial patterns across different marker set sizes.

Next, we compare the results of DECLUST with other deconvolution methods, which are presented in Fig. 4C. In the figure, each row indicates one cell type, while each column refers to a deconvolution method. As shown in the figure, the cell-type proportions identified by both Tangram and Cell2location do not well reflect the expression of the top marker genes in all cell types. CARD, GraphST, and Tangram all perform reasonably well for tumor cells, capturing the expression pattern of the marker gene SLPI. However, compared to DECLUST, Tangram tends to underestimate the overall proportion of tumor cells. For mesangial cells, GraphST and Tangram incorrectly predict elevated proportions on the right side of the tissue, whereas CARD highlights many spots with a high mesangial cell proportion in the bottom-left corner. For chondrocyte cells, Tangram fails to detect this cell type, CARD overestimates their abundance in the bottom-left region, and GraphST underestimates their presence in the same area. Thus, DECLUST preserves better the spatial information of the cell types in the ST data.

### DECLUST exhibits computational efficiency in cell-type deconvolution

**Figure 5.**
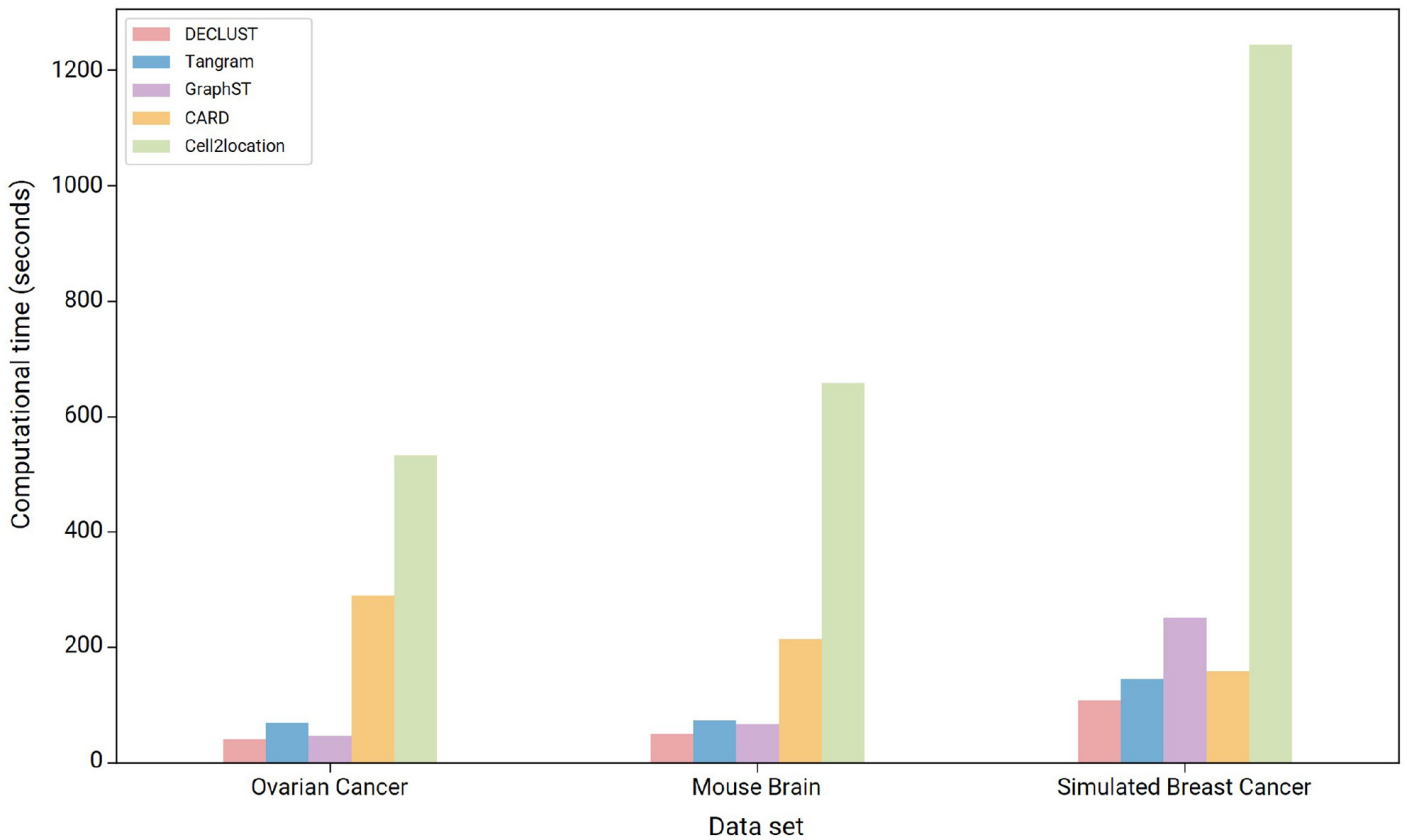
Computational time of the deconvolution methods in three ST datasets. The *x*-axis shows the results of each dataset. The *y*-axis presents computational time in seconds.

To compare the computational time between deconvolution methods, we measure the time starting from data inputting until achieving the cell-type proportions of individual spots. All methods are implemented on a server equipped with an Intel Core i7-12700H CPU and an NVIDIA RTX 3080Ti Laptop GPU. We remove parallel computation for all methods to measure their computational usage. Figure 5 displays the computational time (by seconds) of five methods for one simulated ST dataset and two real ST datasets. The details are provided in Supplementary Table S2. For the real ST datasets, DECLUST, Tangram, and GraphST require relatively short computational time. Although the difference in computational time between DECLUST and GraphST on the real dataset appears modest, we emphasize that DECLUST consistently achieves lower runtime across all datasets. In addition, DECLUST does not rely on GPU acceleration or deep learning frameworks, making it a more lightweight and interpretable solution. Of note, most methods including GraphST, Tangram, and Cell2location require GPUs for their computation. For the simulated ST dataset, Cell2location still has the highest computational cost, while the other methods show favorable computational efficiency.

## Discussion

Recent advances in ST have significantly enhanced our understanding of cell-type composition within tissue architecture [44]. However, due to technical constraints and high costs, most ST data currently lack single-cell resolution, hindering the exploration of the cellular composition at specific tissue locations [45, 46]. We have developed DECLUST, an efficient cluster-based cell-type deconvolution of ST data. We have shown that DECLUST performs well against existing ST deconvolution methods in both simulated and real datasets.

A key advantage of DECLUST lies in its ability to simultaneously cluster spatial data based on both gene expression profiles and spatial coordinates of spots. This spatial clustering approach preserves the spatial structure of the spot clusters. In contrast, conventional clustering methods, such as Seurat [47], and the Louvain method [48] primarily rely on gene expression data alone. As a result, the domains identified by these methods tend to be discontinuous, since they do not incorporate spatial information to recognize co-localized cells that might belong to the same domain. While GraphST improves upon this limitation by introducing spatial proximity through graph-based representation learning, which refines spatial clustering, it still faces challenges. Specifically, smooth boundaries are not always ensured, and the method demands longer computational times due to the need for multiple runs.

Furthermore, by aggregation of gene expression of spots in the same cluster for deconvolution, DECLUST can perform on the higher gene-expression signals as compared to the low gene expression levels of individual spots, consequently resulting in high accuracy. Another unique characteristic of DECLUST is the approach to identify cell-type-specific marker genes. DECLUST aims to detect marker genes from scRNA-seq reference data that their expression is significantly upregulated in one cell type while not statistically different in the remaining cell types. Intuitively, this could decrease the noise for the deconvolution model, subsequently improving the estimation of cell-type composition.

Besides its advantages, DECLUST also has some limitations. For example, similar to other traditional deconvolution methods using the reference from single-cell data, DECLUST cannot estimate the cell types not existing in the single-cell reference. Carefully selecting a good reference data will help to improve the issue. Furthermore, all analyses in this study are based on the ST data generated by 10× Visium, the most commonly used ST technology in the field. Although DECLUST performs well on this type of ST data, it is not clear how it will perform in other ST platforms; this will be worth future studies.

The aggregation can potentially blur fine-grained biological variation across individual spots, and this trade-off between computational robustness and spot-level resolution must be carefully considered. However, the aggregation can still be a correct and biologically reasonable with a proper aggregation strategy to group spots with similar biological profiles together, as what DECLUST is aiming for. In DECLUST, spots are not randomly aggregated, but they are already similar based on both spatial and expression features. Gene expression carries the biological information of spots, but individual spots often suffer from extremely sparse expression in ST. Many genes have zero counts or near-zero, not because they are truly absent, but because of technical limitations such as capture efficiency and sequencing depth. Aggregating across biologically similar spots increases the signal-to-noise ratio, making it possible to detect real patterns. Furthermore, neighboring spots in ST often share highly similar biological profiles, because of, for example, coherent tissue structures such as a cortex layer or tumor clone, etc. Therefore, aggregation of these reflects true biology rather than distorting it. The clustering step with the aggregation strategy contributes significantly to DECLUST’s performance, see Supplementary Fig. S7.

Although the clustering step in DECLUST is currently designed to enhance deconvolution by incorporating spatial coherence, the resulting spatial domains may also serve as a basis for biological interpretation. This is conceptually related to the approach adopted by spacedeconv [49], which utilizes clustering to define and interpret tissue niches. While we have not explicitly assessed the biological relevance of the clusters in this study, future investigations may explore whether the identified clusters reflect tissue microenvironments or spatially organized functional regions. Such exploration could expand the utility of DECLUST beyond deconvolution. Future extensions of DECLUST could also incorporate additional information or modalities beyond gene expression, such as LR interactions, spatially inferred cell–cell communication networks, epigenomic profiles, spatial proteomics, and histology-derived image features. These enhancements may further improve clustering accuracy and help capture complex patterns of intercellular communication and tissue microenvironment heterogeneity.

In summary, we have introduced DECLUST, a highly accurate, efficient, and robust tool for cell-type deconvolution from ST. The results from the simulated and real ST datasets show that DECLUST performs well against other competing methods in both prediction accuracy and preservation of spatial structure in ST data.

## Supplementary Material

gkaf714_Supplemental_File

## Data Availability

This study utilized publicly available datasets, including the Dataset1 (Human Breast Cancer): 10× Visium, Human-Breast-Cancer; 10× Genomics (GSE176078) in the GEO database. Dataset2 (Mouse Brain): 10× Visium, Mouse-Brain-Section; Drop-seq (GSE102827) in the GEO database. Dataset3 (Human Ovarian Cancer): 10× Visium, Human-Ovarian-Cancer; 10× Genomics (GSE192898) in the GEO database.

## Code availability

The DECLUST software is available at https://github.com/Qingyueee/DECLUST and https://doi.org/10.6084/m9.figshare.28945493.
