# Supplementary material for: A cluster-based cell-type deconvolution of spatial transcriptomic data
Source: Nucleic Acids Res. 2025 Jul 24;53(14):gkaf714. doi: 10.1093/nar/gkaf714 (PMC12288882; doi:10.1093/nar/gkaf714)
Supplement: gkaf714_Supplemental_File [file gkaf714_supplemental_file.pdf]

Supplementary information
A clustering-based methodology for cell-type deconvolution in spatial transcriptomic analysis

Qingyue Wang[1,2], Parth Khatri[3], Huy Q. Dinh[3], Jian Huang[1,*], Yudi Pawitan[2,*], Trung Nghia Vu[2,*]

[1]Department of Statistics, University College Cork, Cork, Ireland
[2]Department of Medical Epidemiology and Biostatistics, Karolinska Institutet, Stockholm, Sweden
[3]McArdle Laboratory for Cancer Research, Department of Oncology, School of Medicine and Public Health, University of Wisconsin - Madison, 1111 Highland Ave, WI 53705-227, Wisconsin, USA

[*]Correspondence should be addressed to: trungnghia.vu@ki.se, yudi.pawitan@ki.se, j.huang@ucc.ie

Table S1. Summary of competing cell-type deconvolution methods.

Method	Language	Feature selection	Inference method	Implement
**CARD**	R	Highly variable genes	Non-negative matrix factorization model	CARD deconvolves bulk RNA-seq or spatial transcriptomics data using a non-negative matrix factorization model. It begins by identifying highly variable genes and uses single-cell RNA-seq data as a reference to extract cell-type-specific gene expression profiles. CARD then models the bulk or spatial data as a mixture of these profiles, estimating the proportions of different cell-types in each sample. For spatial data, it incorporates spatial information to improve the accuracy of deconvolution, producing both cell-type proportion estimates and spatial maps of cell-type distributions.
**Cell2location**	Python	No selection	Probabilistic, negative binomial distribution, variational Bayesian inference	Cell2location applies a hierarchical Bayesian framework, assuming gene expression counts follow a negative binomial distribution. It first estimates cell-type-specific signatures using external scRNA-seq data as a reference. The spatial expression count matrix is then modeled with a negative binomial distribution, and variational Bayesian inference is used to approximate the posterior distribution, producing parameter estimates for cell-type proportions accordingly.

**GraphST**	Python	Highly variable genes	Graph self-supervised contrastive learning	GraphST deconvolves spatial transcriptomics data using a graph-based, self-supervised learning framework. It starts by selecting highly variable genes and constructing a spatial graph where nodes represent spatial spots connected based on proximity. Each node is associated with gene expression data. A graph neural network (GNN) is trained using contrastive learning to capture spatial and gene expression patterns. The learned embeddings are then matched to reference single-cell RNA-seq data to estimate cell-type proportions in each spot, providing both deconvolved cell-type distributions and low-dimensional representations of the data.
**Tangram**	Python	Highly variable genes	Deep learning model	Tangram takes sc/snRNA-seq and spatial transcriptome data from the same region or tissue, requiring only a shared subset of common genes. It initially places sc/snRNA-seq expression profiles randomly in space, then computes an objective function to model the spatial correlation between genes in both datasets. Tangram iteratively rearranges the sc/snRNA-seq profiles to maximize the total spatial correlation of the shared genes.

Table S2. Comparison of five deconvolution methods by computational time (seconds).

	DECLUST	CARD	Cell2location	Tangram	GraphST
**Breast Cancer**	108.16	158.50	1242.88	145.13	251.25
**Mouse Brain**	50.39	214.75	657.82	73.26	67.65
**Ovarian Cancer**	41.19	289.75	532.25	69.82	46.96

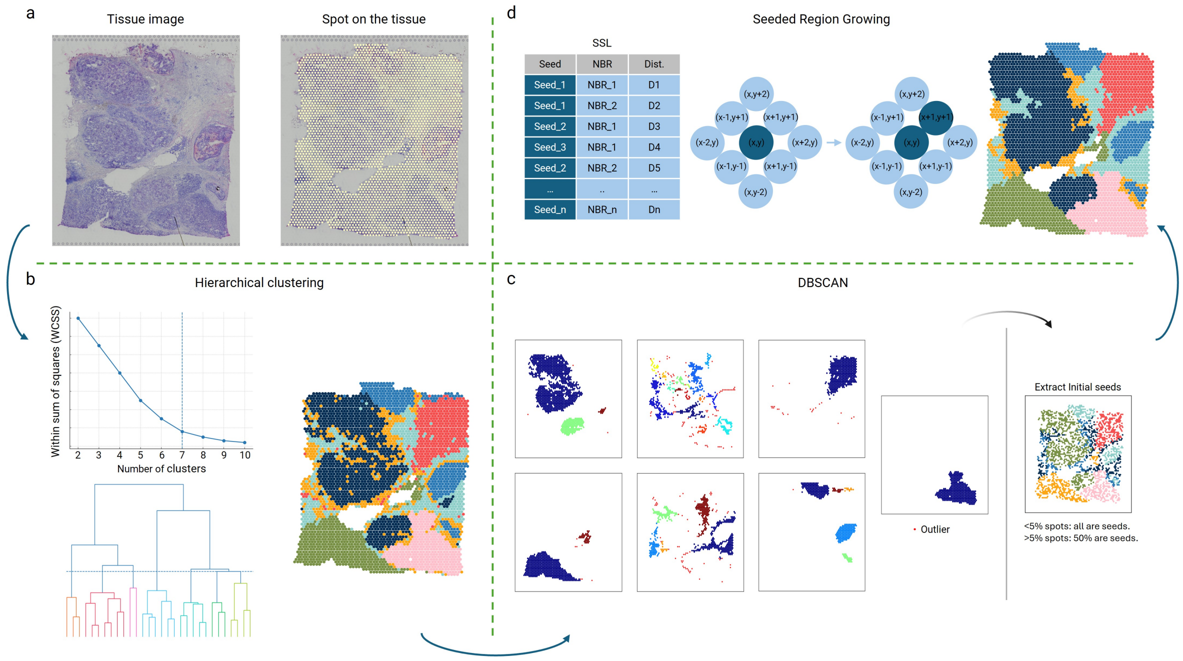

Figure S1. Overview of the proposed clustering approach of DECLUST. (a) The tissue images and the corresponding under-tissue spots are shown, serving as the starting spot for the analysis. (b) Hierarchical clustering is applied to the gene expression data to generate initial clusters, with the optimal number of clusters determined using the elbow method, as demonstrated by the within-cluster sum of squares (WCSS) plot. (c) DBSCAN (density-based spatial clustering of applications with noise) is applied to the initial clusters to identify spatial sub-clusters and outliers. (d) The seeded region growing (SRG) algorithm is used to refine the clusters, taking into account both gene expression similarity and spatial proximity of spots. Seeds are selected based on sub-cluster size and input into the SRG to finalize spot clusters, producing a segmentation that integrates both spatial and gene expression features.

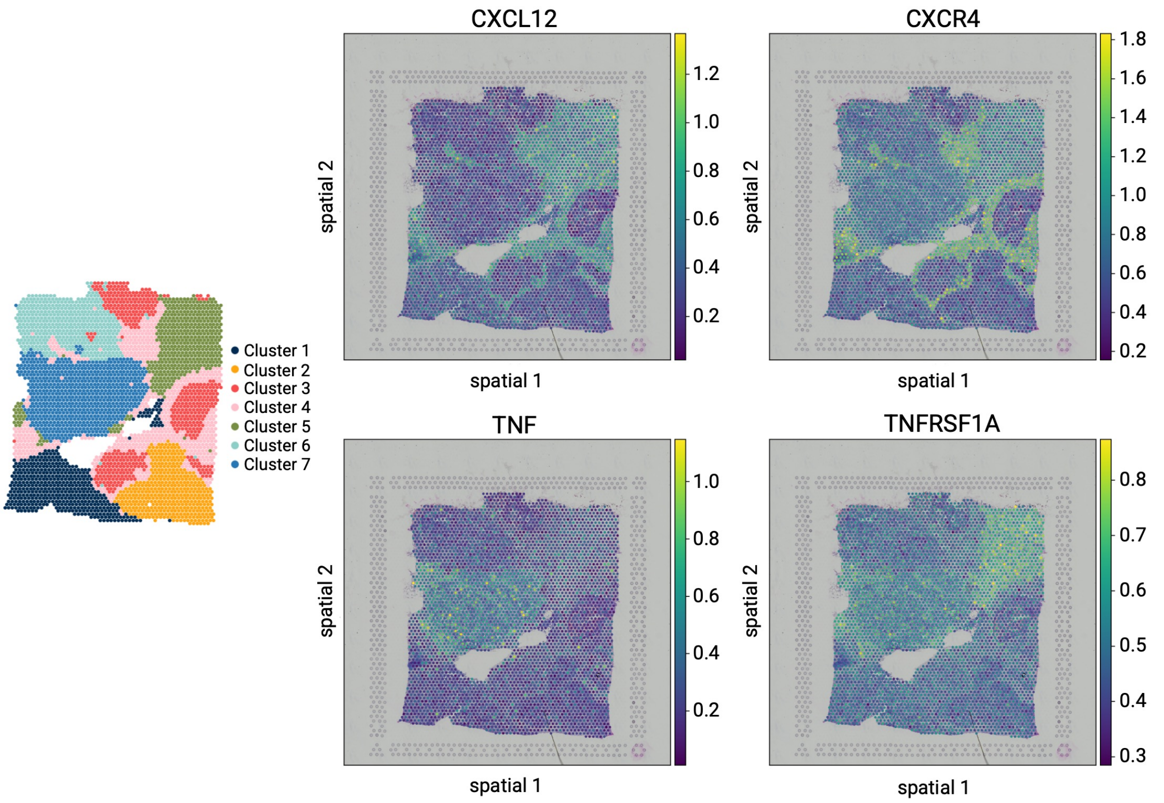

Figure S2. Spatial expression of Ligand-Receptor pairs used to guide cell-type enrichment in Simulation 2. The left panel displays the spatial clusters identified by BayesSpace in the simulated dataset, with each color representing a distinct cluster (Clusters 1–7). The right panels show the spatial expression patterns of four selected ligand-receptor genes: CXCL12, CXCR4, TNF, and TNFRSF1A. The ligand-receptor pair CXCL12–CXCR4 guides the enrichment of CAF and Plasmablast cells in Cluster 4, while TNF–TNFRSF1A guides the enrichment of Myeloid and Endothelial cells in Cluster 7. These spatial plots illustrate the localized expression patterns that support the targeted enrichment strategy.

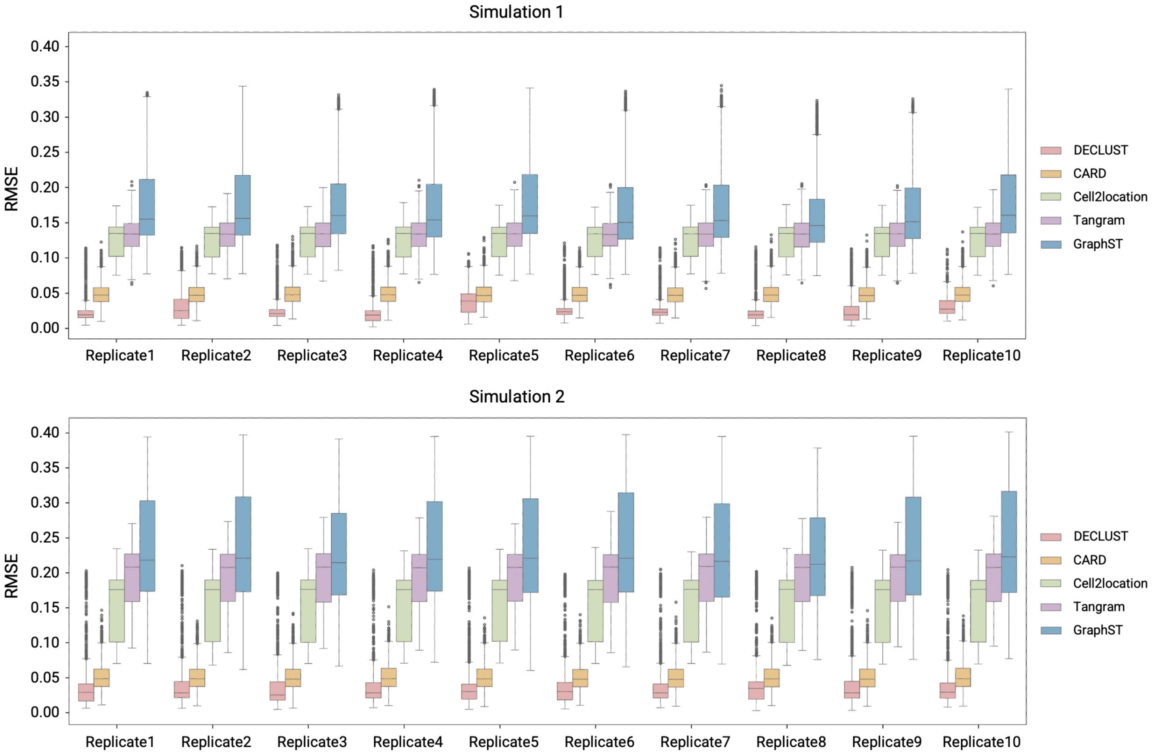

Figure S3. Comparative performance of spatial transcriptomics methods under two simulation settings across 10 replicated datasets in Simulation 1 and Simulation 2. Boxplots display the distribution of root mean square error (RMSE) for five deconvolution methods—DECLUST, CARD, Cell2location, Tangram, and GraphST—across 10 independent replicates for each simulation scenario. In both Simulation 1 and Simulation 2, DECLUST consistently demonstrates the lowest median RMSE values, with narrow interquartile ranges and fewer outliers, suggesting both high accuracy and robustness. CARD follows with slightly higher RMSEs but remains consistently lower than Cell2location, Tangram, and GraphST. Notably, GraphST shows the highest RMSE and largest variability across replicates, with median values exceeding 0.20 in most cases. These results indicate that DECLUST significantly outperforms existing methods in terms of deconvolution accuracy and consistency across diverse simulated conditions.

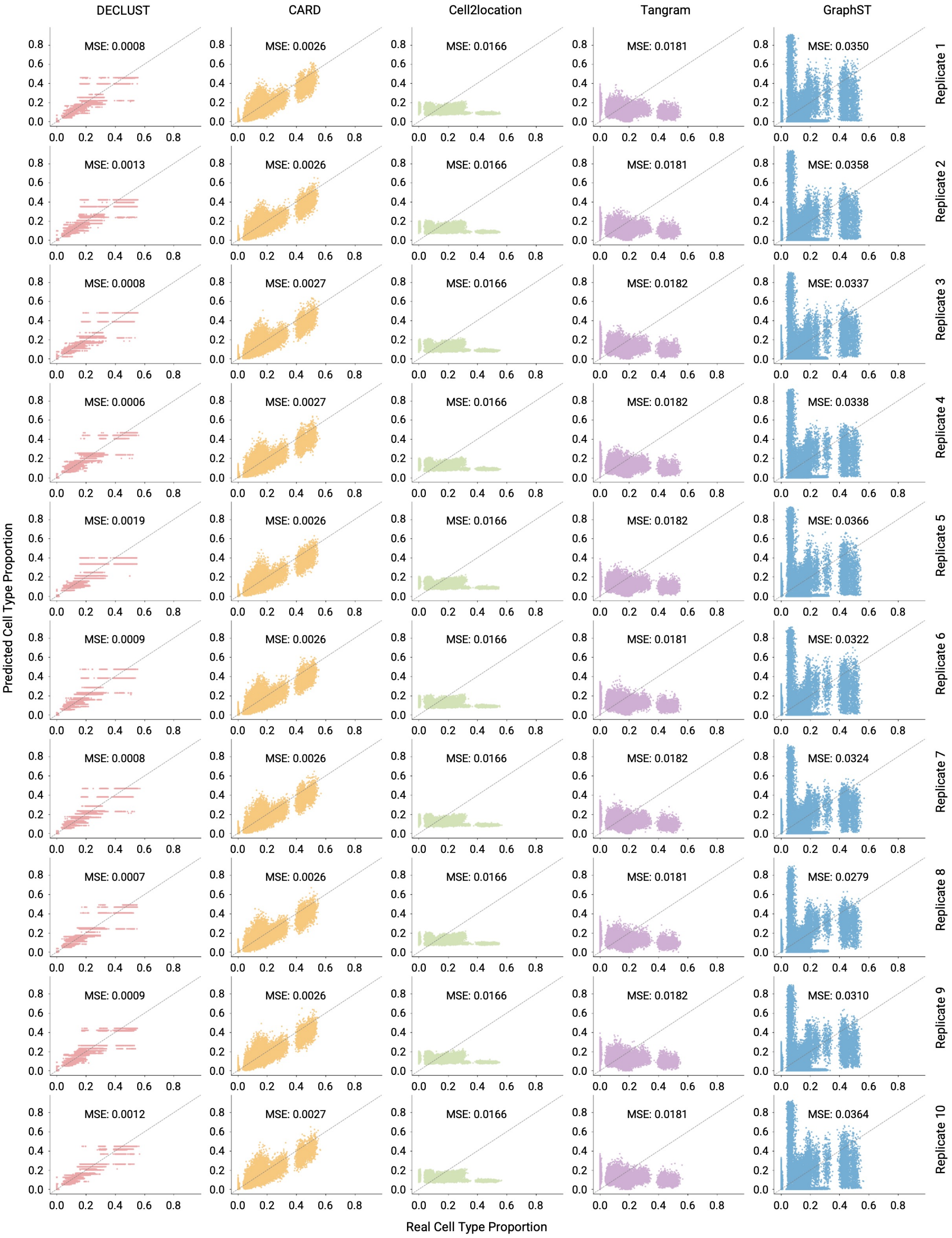

Figure S4. Accuracy of cell-type proportion predictions across multiple methods in Simulation 1. Each scatter-plot compares the predicted and true cell type proportions for DECLUST, CARD, Cell2location, Tangram, and

GraphST across 10 independent replicates under the Simulation 1 framework. Points closer to the diagonal line indicate more accurate predictions. DECLUST achieves the highest agreement with the ground truth, with mean squared error (MSE) values ranging from 0.0006 to 0.0019, indicating exceptional precision. CARD also performs well, with consistent MSEs around 0.0026–0.0027. In contrast, Cell2location and Tangram show moderate performance, each producing stable but higher MSEs (0.0166 and 0.0181, respectively). GraphST displays the largest prediction error and spread, with MSEs ranging from 0.0279 to 0.0366 and predictions frequently diverging from the diagonal. These results underscore the superior accuracy of DECLUST in settings that align with its modeling assumptions, highlighting its strength in capturing true cell type proportions under idealized simulation conditions.

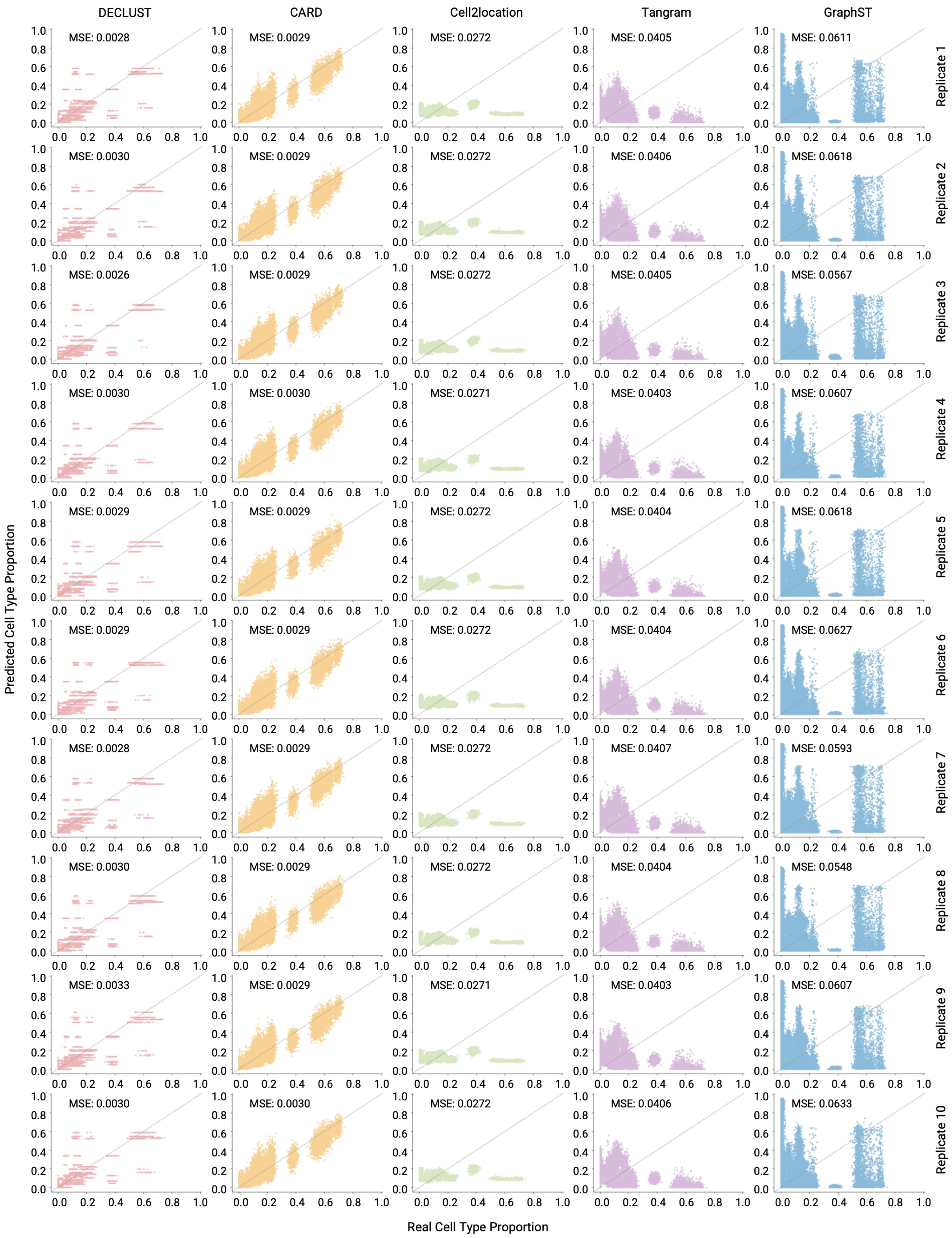

Figure S5. Accuracy of cell-type proportion predictions across multiple methods in Simulation 2. Scatterplots compare the predicted and true cell type proportions for five deconvolution methods—DECLUST, CARD, Cell2location,

Tangram, and GraphST—across 10 replicates datasets in the Simulation 2 setting. Each point represents a capture spot, and the dashed diagonal line indicates perfect agreement between predicted and real proportions. DECLUST and CARD show close alignment with the diagonal, with notably low mean squared error (MSE $\approx$ 0.0028–0.0033), indicating high accuracy in cell type prediction. Cell2location and Tangram exhibit moderate deviations, with MSE values around 0.0272 and 0.0404, respectively. GraphST displays the largest discrepancies, with MSEs frequently exceeding 0.06 and greater dispersion from the diagonal. These results further confirm the strong predictive performance of DECLUST under Simulation 2, even in a more challenging scenario with alternative assumptions.

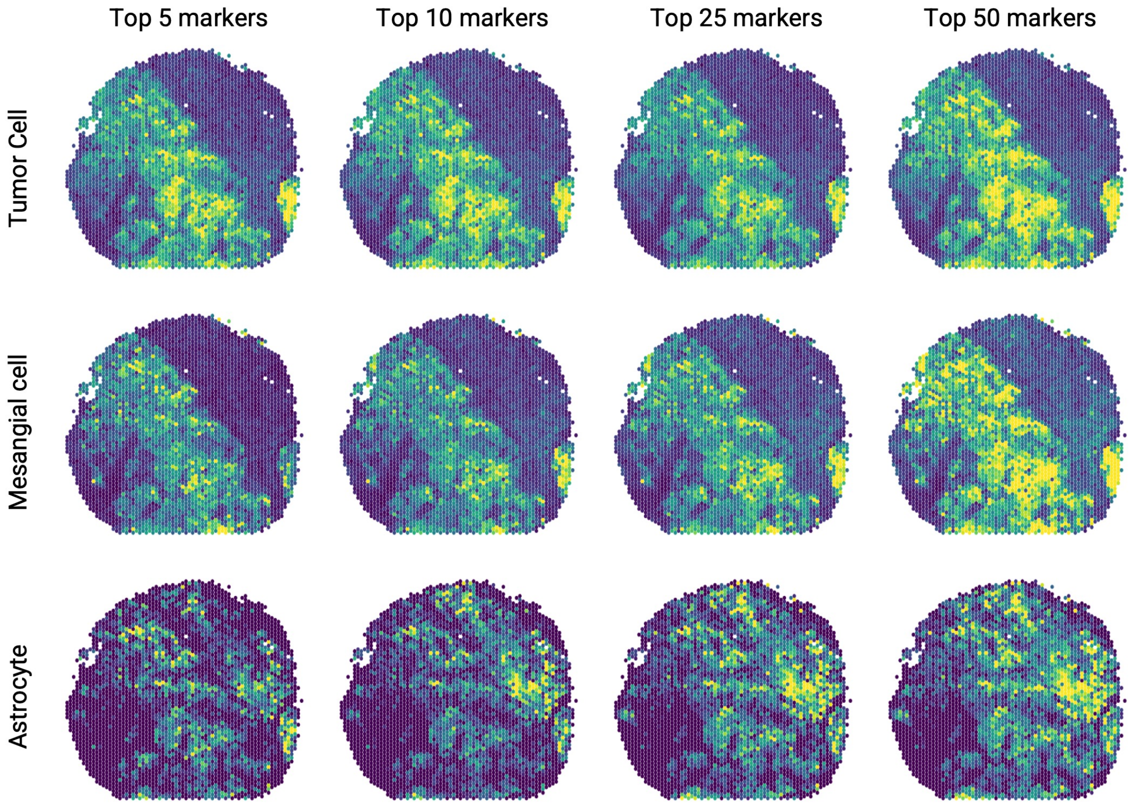

Figure S6. Spatial expression patterns of marker genes for selected cell types across varying marker set sizes. Heatmaps display the aggregated spatial expression of the top 5, 10, 25, and 50 marker genes for three representative cell types—tumor cells, mesangial cells, and chondrocyte—derived from real spatial transcriptomics (ST) data. Marker genes were ranked and selected based on the t-statistic, which measures differential expression between the target cell type and others. For each marker set size, normalized expression values of the selected genes were summed across spatial spots to produce a composite heatmap. The resulting spatial patterns are consistent across different numbers of markers, demonstrating coherent and robust localization signals for each cell type.

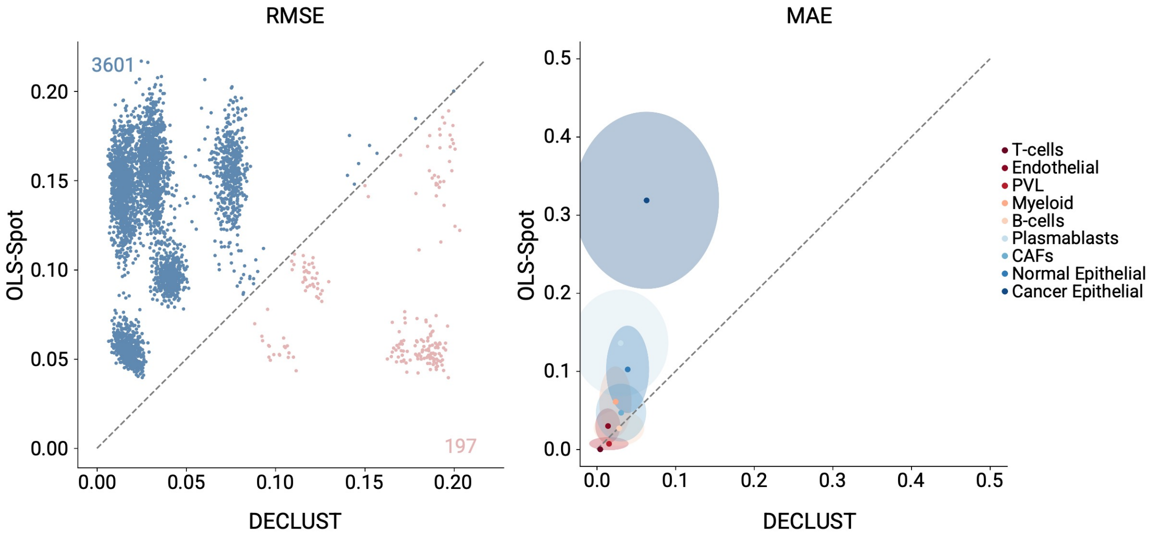

Figure S7. Evaluating the contribution of the clustering step in DECLUST. Scatter plots illustrating the performance comparison between DECLUST and OLS deconvolution on simulated dataset 1. Left: Root Mean Squared Error (RMSE) per spatial spot. Each point represents a single spot, with the x-axis showing the error from DECLUST and the y-axis showing the error from OLS. The majority of points lie above the diagonal, indicating that DECLUST achieves lower RMSE than OLS on 3601 out of 3798 spots. This suggests that incorporating the clustering step before deconvolution consistently improves prediction accuracy at the spot level. Right: Mean Absolute Error (MAE) per cell type. Each point represents the average MAE for a specific cell type, and the size of each ellipse corresponds to the variability across spots. Most points fall below the diagonal, indicating lower MAE for DECLUST compared to OLS. Notably, larger improvements are observed for cell types with higher variability under OLS, such as Cancer Epithelial and Normal Epithelial cells, highlighting the robustness of the clustering-enhanced approach in handling heterogeneous populations.
